# Design and Characterization of PLA Bilayer Films Containing Lignin and Cellulose Nanostructures in Combination With Umbelliferone as Active Ingredient

**DOI:** 10.3389/fchem.2019.00157

**Published:** 2019-03-26

**Authors:** Magdalena L. Iglesias Montes, Francesca Luzi, Franco Dominici, Luigi Torre, Viviana P. Cyras, Liliana B. Manfredi, Debora Puglia

**Affiliations:** ^1^Facultad de Ingeniería, Instituto de Investigaciones en Ciencia y Tecnología de Materiales, Universidad Nacional de Mar del Plata, Mar del Plata, Argentina; ^2^Civil and Environmental Engineering Department, UdR INSTM, University of Perugia, Terni, Italy

**Keywords:** poly (lactic acid), cellulose nanocrystals, lignin nanoparticles, umbelliferone, melt-compounding techniques, mechanical properties, food packaging

## Abstract

Poly (lactic acid) (PLA) bilayer films, containing cellulose nanocrystals (CNC) or lignin nanoparticles (LNP) and *Umbelliferone* (UMB) were extruded and successfully layered by thermo-compression starting from monolayer films. Lignocellulosic nanostructures were used in PLA based film as nanofillers at 3 wt.%, while UMB was used as active ingredient (AI) at 15 wt.%. The effects of processing techniques, presence, typology and content of lignocellulosic nanoparticles have been analyzed and thermal, morphological, mechanical and optical characterization of PLA nanocomposites have been made. Furthermore, X-ray diffraction (XRD) and Fourier Transform Infrared spectroscopy (FTIR) studies evaluated the presence of nanofillers and AI at chemical level. Bilayer formulations showed a good interfacial adhesion and improved stress at break with respect of PLA monolayers, although they were less stretchable and transparent. Data obtained from thermal, colorimetric and transparency investigations underlined that the presence of lignocellulosic nanofillers and AI in PLA monolayer and bilayer films induced relevant alterations in terms of overall color properties and thermal stability, while antioxidant activity of umbelliferone was enhanced by the addition of lignin in produced materials.

## Introduction

The continue demand of semi prepared natural and fresh food products with high nutritional value have promoted the current trend in food packaging application to develop new systems to improve the shelf-life of packaged foods (Fabra et al., 2016; Luzi et al., 2018b). Specifically, the packages contain, cover and protect the foodstuffs from the external attacks as light irradiation, external contaminations, and oxidation induced by the contact with the air (Lee et al., 2015; Domínguez et al., 2018; Luzi et al., 2018a). The shelf-life of certain foods may be significantly limited if no some chemical ingredients are added. The meat is one of the main perishable food characterized by the presence of fat and a high content of unsaturated fatty acids subjected to lipid oxidation causing the quality deterioration in meat and meat based products and consequentially to deterioration process was observed also the microbial growth (Yang et al., 2016a; Banerjee et al., 2017; Domínguez et al., 2018).

In this scenario, several strategies were adopted to improve the quality and the safety of packaged foods, to reduce the environmental burden due to the disposal of spoiled foods, to increase the quantity of food available for human utilization and finally to reduce the economic impact caused by the deteriorations of foods (Yang et al., 2016a). The different measures adopted on food preservation/protection play a great function. Food conservation consists in preventing all aspects concerned in food contamination and deterioration which are the main problem of loss in safety of food and nutritional value. The most important are the oxidative processes, which are liable for the modification of flavor, the degradation of vitamins, and the deterioration of foodstuffs such as the browning of meat, fruits and vegetables. Active packages symbolize some interesting possibilities spanning through the entire food chain from producers and distributors to consumers (Lee et al., 2015).

Recent research studies proposed some different approaches to modulate and to enhance the shelf-life of foods: incorporation of active ingredients/agents into packaging based systems and the development of bilayer polymeric based systems to modulate the exchange of gas flow between the internal and external parts of the packaging (Muller et al., 2017). Recently, several phenolic compounds and natural extracts as hydroxytyrosol, rosmery, carvacrol, catechin, gallic acid, etc. (Cerruti et al., 2009; Castro López et al., 2013; Arrieta et al., 2016; Fortunati et al., 2016a; Luzi et al., 2017, 2018a; Piñeros-Hernandez et al., 2017) have been studied in combination with polymeric materials to improve the active properties of neat polymers of great significance in the food sector.

Antioxidant active and smart packaging seeks to stop or reduce the oxidation of different food components, principally, proteins and lipids, which lead to the deterioration of physical and organoleptic properties (such as color and flavor). The realization of active polymeric based materials is centered on the incorporation of antioxidant compounds into the packages and their further migration to the food (Fabra et al., 2016). Polymers, and specifically biodegradable polymers, are the main preferred materials for food packaging application. In fact, green based polymeric systems represent an alternative to develop novel eco-friendly systems that can reduce the amount of wastes in landfills respect to petroleum based polymers. Respect to traditional polymers green based systems can also minimize the greenhouse gas emissions during the realization and the end-of-life disposal (Peelman et al., 2013).

In spite that usually green polymers have lower mechanical performance and poorer barrier properties than traditional ones, they are feasible materials for active packaging due to their intrinsic characteristics. They can be good carrier substrates of active principles because of their adaptability to controlled release and also they can be combined through the realization of polymeric blends or multilayer formulations in order to optimize the application (Martínez-Abad et al., 2014). In this context, the development of bilayer films represent a strategic alternative to improve the barrier essential in the fresh food package to guarantee the shelf-life of the products and the mechanical properties of biobased systems (Muller et al., 2017).

Poly(lactic acid) (PLA) is a thermoplastic linear aliphatic polyester, obtained from renewable resources, compostable and biodegradable (Chaiwutthinan et al., 2015). PLA is accepted by US Food and Drug Administration (FDA) as a food contact matter, non-toxic or carcinogenetic, easy to process, transparent, economically feasible, and it is utilized to realize packaging for short shelf-life uses (Arrieta et al., 2013). Unluckily, as described in general for biodegradable polymers also PLA is characterized by some drawbacks when compared with petroleum base matrices utilized in food packaging sector.

Nanocomposites represent as a valid strategy to modulate the moderate characteristics of biodegradable polymers. Recently, lignin nanoparticles (LNPs) and cellulose nanocrystals (CNC) have been used as nanoreinforcements to enhance the characteristics of biobased polymeric systems for food packaging applications (Fortunati et al., 2013, 2016b; El-Wakil et al., 2015).

Lignin components provide specific food antioxidant properties (Domenek et al., 2013; Yang et al., 2016b) and UV stabilization effect (Chung et al., 2013). It has been established that the radical scavenging activity is related to particle size and that the antioxidant effect increase decreasing the dimensions of fillers (Ge et al., 2014). Moreover, enhanced thermal and mechanical characteristics of different polymer matrices were found (Nair et al., 2014). Additionally, LNPs is a promising green agent useful against dangerous bacteria and microorganisms, its intrinsic biocide effect allows to decrease the environmental problems associated to the utilize of silver nanoparticles (Richter et al., 2015; Yang et al., 2016b).

With the same aim, cellulose nanocrystals (CNC), which are characterized by low density, high biocompatibility and stiffness (Fernandes et al., 2013), contribute to increase the overall thermomechanical performance of polymeric materials, due to the synergic interactions of crystal nucleation, tortuosity and chain immobilization (Pracella et al., 2014; Kamal and Khoshkava, 2015). Moreover, active ingredients such as umbelliferone (7-hydroxycoumarin), a natural phenolic extract extensively spread in plants of the coumarin family with antioxidant and antimicrobial effects (Mazimba, 2017; Luzi et al., 2018b), could improve the antioxidant response of polymeric films even in a multilayer approach. While the use of natural, non-toxic antioxidants such as ferulic acid or α-tocopherol to extend the food shelf life has been investigated, limited information exists about the influence of umbelliferone on films structure and physicochemical properties in packaging application. The successful use of UMB in EVOH matrix has been already tested and compared with gallic acid effectiveness in Luzi et al. (2018b). In addition, on the basis of available relevant results on UMB use in medicine (umbelliferone has been demonstrated to have biological activity as an antibacterial, anti-inflammatory, antirheumatic, and immunomodulatory agent), we planned to comprehensively verify its possible use in a sector having similar requirements, to demonstrate enhanced antibacterial and antioxidant properties when combined with lignin based nanoparticles.

The aim of this work is therefore the design, realization, and characterization of poly (lactic acid) (PLA) based bilayer films for food packaging applications. PLA monolayer films containing cellulose nanocrystals (CNC) or lignin nanoparticles (LNP) and Umbelliferone (UMB) were extruded and successfully layered by thermo-compression. The effect of the different nanoparticles and the active ingredient (UMB) on the properties of the bilayer system was studied. Emphasis was placed on the determination of antioxidant and light transmission results according to the proposed use of the materials as packaging.

## Materials and Methods

### Materials

Poly(lactic acid) (PLA) 3051D, with a specific gravity of 1.25 g cm^−3^, a molecular weight (*M*_*n*_) of ca. 1.42 × 10^4^ g mol^−1^, and a melt flow index (MFI) of 7.75 g 10 min^−1^ (210°C, 2.16 kg) was purchased by Nature Works®, USA. Pristine lignin was supplied by CRB (Centro Ricerca Biomasse, University of Perugia) and lignin nanoparticles (LNP) were synthesized as previously reported (Yang et al., 2015a). Microcrystalline cellulose (MCC, dimensions of 10–15 μm), utilized as cellulose nanocrystals precursor during the hydrolysis process, was purchased by Sigma-Aldrich®. Cellulose nanocrystals (CNC) were synthesized as previously reported (Fortunati et al., 2014). Umbelliferone (UMB) and all the chemical reagents were purchased from Sigma Aldrich^®^ and used as received.

### Preparation of PLA Nanocomposite Films

PLA pellets, CNC and LNP were previously dried in an air circulating oven at 50°C overnight in order to eliminate moisture traces. Neat PLA, two different formulations of PLA nanocomposite (PLA_3%CNC, PLA_3%LNP), and a PLA active film (PLA_15%UMB) were realized in a twin screw microextruder (DSM Explore 5&15 CC Micro Compounder) taking as a reference sample neat PLA. The processing parameters were optimized for each formulation. During the mixing, the screw speed was set at 100 rpm through 3 min (with nano-compound it was 2 min for the neat PLA and 1 min for de nano-component), with a temperature profile of 180–190–200°C throughout the three zones and a dye temperature of 195°C. The films obtained following this process have a nominal thickness between 80 and 85 μm.

### Preparation of Bilayer Films

In order to obtain bilayer films, the different PLA monolayers were assembled and compressed at 155°C for 1 min at a pressure of 25 bar followed by a cooling cycle. Six kinds of films were obtained: pure polymer bilayer film (PLA/PLA), as a control, and films with nano and active compounds (PLA/PLA_3%CNC, PLA/PLA_3%LNP, PLA/PLA_15%UMB, PLA_3%CNC/PLA_15%UMB, PLA_3%LNP/PLA_15%UMB). CNC, LNP and UMB have been selected according to previous results obtained by the authors on separated or combined use of CNC and LNP in polylactic acid (Yang et al., 2016b), while the amount of UMB was selected on the basis of dispersability results and its effect of transparency and antibacterial response when introduced in polymeric film formulations (Luzi et al., 2018b).

### Film Characterization

#### Structural Characterization

X-ray diffraction (XRD) analysis of the samples was obtained with an X-Pert pro diffractometer with CuKα radiation (λ = 1.54 Å), operating at 40 kV and 40 mA. The diffraction profile was detected from 4 to 60° at a scanning rate of 2°/min.

FT-IR measurements were performed at room temperature in reflection mode in attenuated total reflectance (ATR) using a Thermo Scientific Nicolet Instrument 6700. Spectra were acquired within 4,000–600 cm^−1^ region, using 32 scans overlapped and 4 cm^−1^ resolution.

The cross section micromorphology of the films was observed using a field emission scanning electron microscope (FESEM, Supra 25- Zeiss, Germany). Films were initially fractured in liquid nitrogen and gold coated with an Agar automatic sputter coated.

#### Light Transmission and Color Properties

The light transmission test were performed on film samples in the visible light region (400–700 nm) using a spectrophotometer (CM-2300d Konica Minolta, Japan), while film color properties were evaluated by using the CIELAB color space by means Multi-Gloss 268, Minolta, Langenhagen, Germany. Color coordinates, L^*^ (lightness), a^*^ (red–green), and b^*^ (yellow– blue) were measured. The instrument was calibrated using a white standard tile. Average values of three different measurements at random positions over the film surfaces were obtained. Total color difference (ΔE^*^) was evaluated with respect to the white control as follow:

(1) $$\begin{array}{r} \Delta E^{*}=\sqrt{{\left(\Delta L^{*}\right)}^{2}+{\left(\Delta a^{*}\right)}^{2}+{\left(\Delta b^{*}\right)}^{2}} \end{array}$$

Gloss value was also evaluated using a flat surface gloss meter at an incidence angle of 60°, according to the ASTM standard D523 (ASTM, 2018).

#### Thermal Properties

##### Differential scanning calorimetry (DSC)

DSC analysis was performed by using a TA Instruments DSC Q200 in modulated mode (TA Instruments Inc., USA) under nitrogen atmosphere. The scanning process covered from −25 to 210°C, applying two heating and one cooling scan, at 10°C/min. Glass transition, crystallization and melting phenomena of film samples were defined from the first heating scan. Degree of crystallinity (*Xc*) was evaluated by using Equation (2):

(2) $$\begin{array}{r} \chi_{C}=\left(\frac{\Delta H_{m}-\Delta H_{cc}}{w\cdot \Delta H_{m}^{0}}\right)\times 100 \end{array}$$

where Δ*H*_*m*_ and Δ*H*_*cc*_ are the enthalpy of melting and cold crystallization peaks, respectively; *w* is the weight fraction of PLA polymer in the sample and Δ*H*${}_{m}^{0}$ is the melting enthalpy of PLA 100% crystalline (93 J/g) (Abdelwahab et al., 2012).

##### Thermogravimetric analysis (TGA)

Dynamic thermal degradation was performed using a TG-50 Shimadzu, under nitrogen atmosphere. Temperature was raised from 25 to 800°, at a heating rate of 10°C/min. The initial degradation temperature (*T*_0_) was calculated at 5% mass loss, while temperatures at the maximum degradation rate (*T*_*max*_) for each stage were determined from the first derivatives of the TGA curves (DTG).

#### Mechanical Properties

Tensile characteristics of different formulations were evaluated at room temperature using an universal testing machine, based on UNI ISO 527 standard (crosshead speed of 1 mm/min). Mechanical parameters, i.e., elastic modulus (EM), tensile strength (TS), and elongation at break (ε), were obtained from the resulting stress–strain curves. Reported values were the average of at least five valid tests.

#### Antioxidant Activity

The antioxidant activity of films samples was evaluated using a spectroscopic method based on the disappearance of the absorption band at 517 nm of the free radical 2,2-diphenyl-1-picrylhydrazyl (DPPH) (Sigma-Aldrich®), upon reduction by an antiradical compound.

The radical scavenging activity was determined by using a spectroscopic method according to the method reported in literature by Byun et al. (2010). The different systems (0.1 g) were cut into small pieces and immersed in 2 mL of methanol for 24 h. An aliquot of methanol extract (1 mL) was uniformly mixed with 1 mL of DPPH in methanol (50 mg L^−1^). The mixture was allowed to stand at RT in the dark for 60 min. The absorbance was measured at 517 nm using a UV spectrometer (Lambda 35). The DPPH mixture solution of methanol obtained from neat PLA systems was utilized as control. DPPH radical scavenging activity (RSA) was calculated according to the Equation (3):

(3) $$\begin{array}{r} \left(RSA,\%\right)=\left[\frac{A_{control}-A_{sample}}{A_{comtrol}}\right]\times 100 \end{array}$$

where A_control_ is the absorbance of the control (methanol) at t = 0 min and A_sample_ is the absorbance of tested sample after 1 h of incubation.

## Results and Discussion

### XRD Characterization

**Figure 1:**
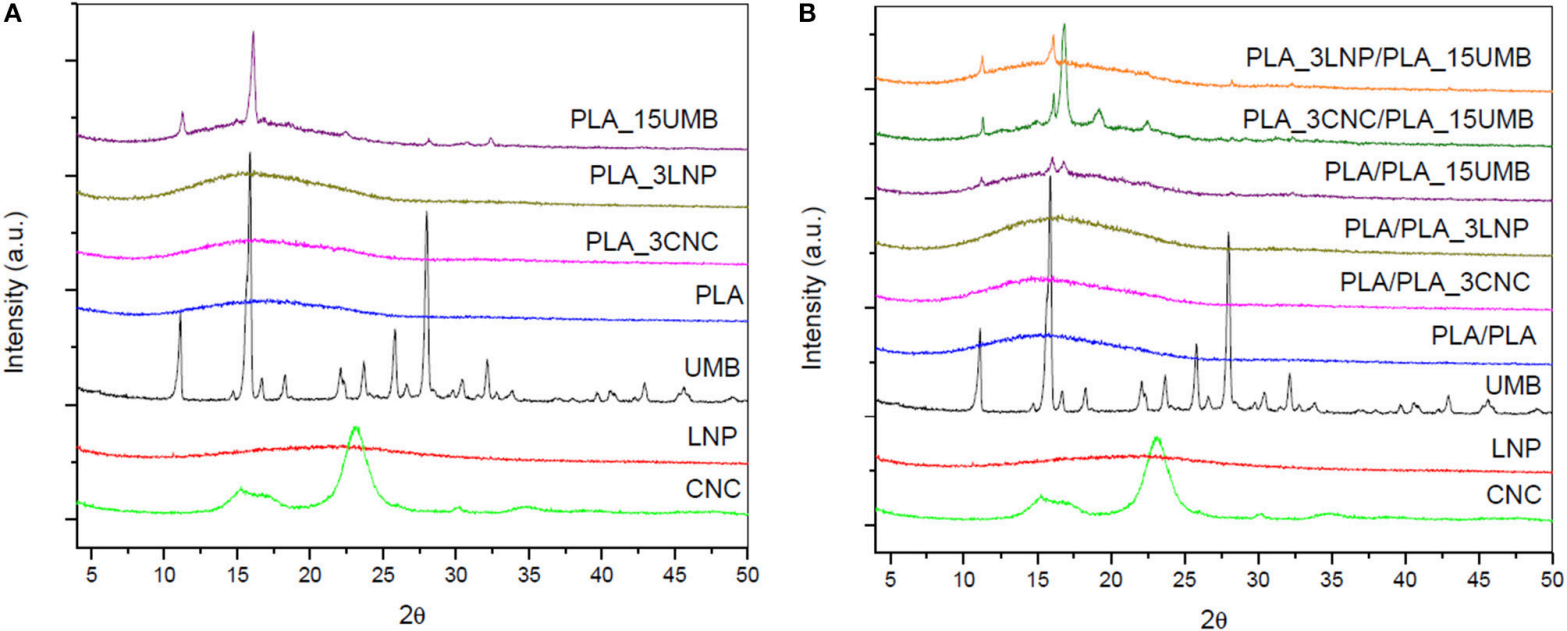
XRD spectra of PLA and PLA nanocomposites **(A)** monolayer films; **(B)** bilayer films.

In Figure 1, X-ray diffraction patterns of different components, monolayer (Figure 1A) and bilayer PLA films (Figure 1B) are reported. In order to study the effect of the different ingredients on the crystalline structure of the PLA, XRD traces for cellulose nanocrystals, lignin nanoparticles and umbelliferone have been also included. The crystalline structure of cellulose nanocrystals was characterized by four main reflection peaks located at 2θ = 15.08, 16.38, 22.58, and 34.5 (Ludueña et al., 2015), while XRD pattern of lignin nanoparticles, that consists of phenylpropane unit forming a three dimensional polymer network without an ordered and regular super-molecular structure, shows a broad peak, indicating that the LNPs were only partially crystalline in nature (Gupta et al., 2014). Umbelliferone showed main peaks at 2θ = 11.2, 16.0, 25.9, and 28.1 (Liu et al., 2017).

In the case of PLA monolayers, it was observed that crystallization was not induced by the addition of any nanoadditive, since no additional peaks appeared in the spectra of the monolayers. Neat PLA presented a halo centered at 2θ = 16°, which is characteristic of amorphous scattering of PLA (Abdelwahab et al., 2012), as already observed by Gorrasi and Pantani (2013). The XRD profile was not modified in presence of CNC and LNP nanofillers, while main characteristic peaks of umbelliferone were detected in PLA_15UMB at 11.3, 16.1, and 28.2°.

No apparent change in crystallinity, as a result of the introduction of lignin and cellulose as nucleating agent, was evidenced in the X-ray diffractograms of PLA bilayers: a similar amorphous band as that identified in the PLA monolayer, regardless of the adhesion to the single or composite PLA layer, was detected, while the signal of umbelliferone, present in higher amount with respect of CNC and LNP, was more visible.

Actually, XRD patterns of PLA bilayers showed some dissimilarity with respect to monolayer PLA films in the case of PLA_3CNC/PLA_15UMB, highlighting the influence the different crystalline phases. Indeed, a new peak at 2θ = 19.2° was recorded, revealing the presence of α′-form crystals (Kalish et al., 2011). Moreover, the diffraction shoulders located at 14.9 and 16.8° may be owed to low crystallinity of the PLA and its imperfect crystals (Jia et al., 2017). These results suggest that the presence of cellulose nanocrystals and diffusion of UMB during thermo-compression slightly influenced the crystallization pattern of PLA due to the promotion of chain interactions with these compounds.

### FTIR Analysis

**Figure 2:**
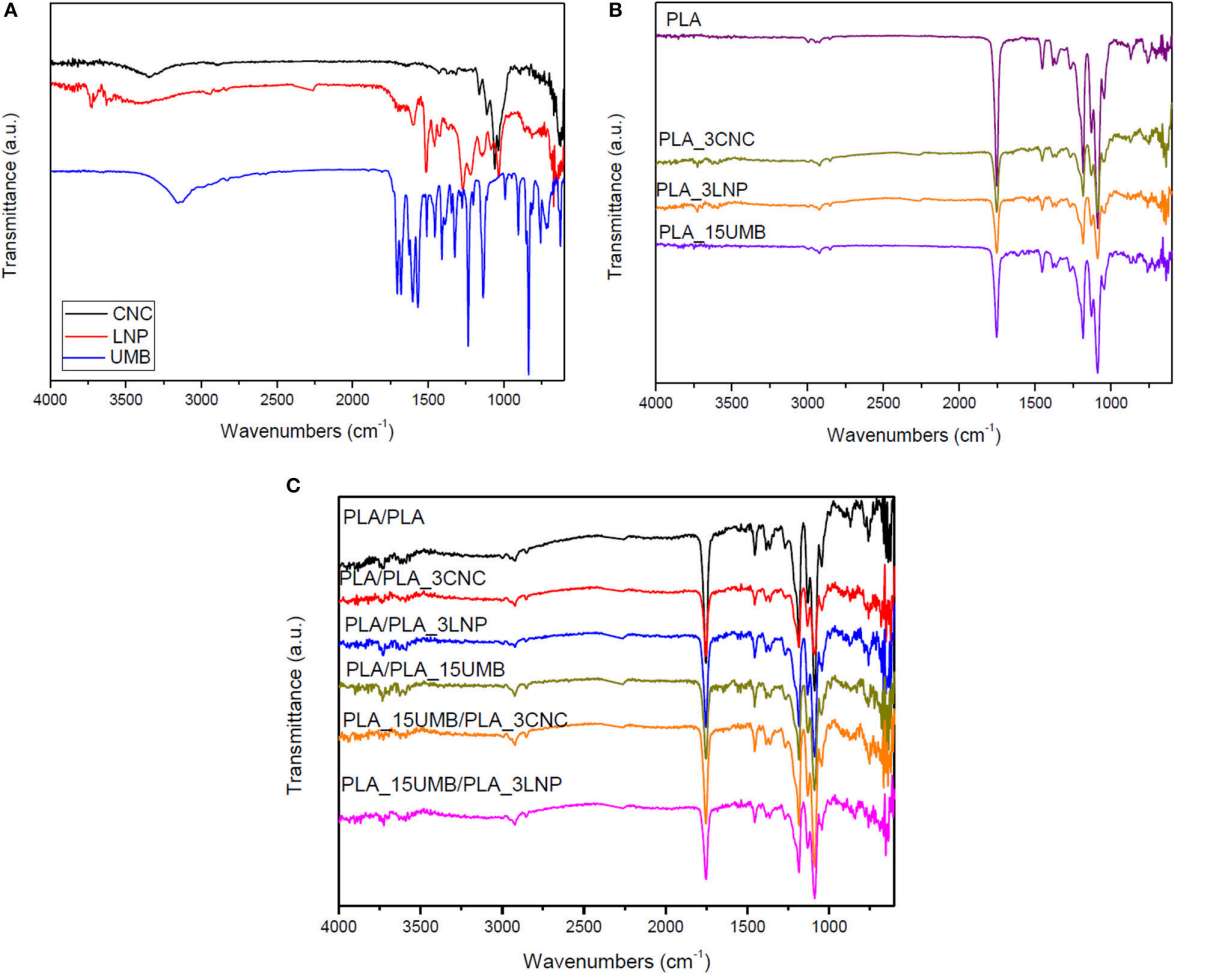
FTIR spectra of nanofillers (CNC and LNP) and AI **(A)**, PLA and PLA nanocomposites monolayer films **(B)**, and bilayer films **(C)**.

FTIR analysis was performed on powders (Figure 2A), monolayer (Figure 2B), and bilayer films (Figure 2C) in order to identify the influence of the additives on the chemical structure of the PLA. The FT-IR spectrum of umbelliferone showed a sharp peak of OH group at 3,153 cm^−1^ (Ittadwar and Puranik, 2017), frequencies identified at 3,122 and 2,987 cm^−1^ are designated to C-H stretching vibrations, single bonded carbonyl group vibrations at 1388, 1,345, and 1,233 cm^−1^, while frequencies at 1,578, 1,454, 1,446, 1,409, and 1,386 cm^−1^ have been designated to C-C stretching vibrations (Rexali, 2007).

Lignin spectrum show complex bands and this is due to the variety of vibration mode of chemical bonds present in this biopolymer structure. The aromatic skeleton vibrations in lignin nanoparticles are assigned at 1,540 cm^−1^ (C–C stretching of aromatic skeletal around), C–H stretching at 1,480 cm^−1^ and CH vibration of methyl group at 1,440 cm^−1^ (Yang et al., 2015b). CNC infrared spectrum was characterized by the presence of the absorption peaks around 3,330, 2,897, 1,654, and 1,025 cm^−1^ related, respectively, to O–H, C–H stretching, C–C bending, and C–O stretching vibrations of cellulose (Bassett et al., 1963).

The main characteristic peaks of PLA were also identified in the spectra showed in Figure 2B. The main one at 1,750 cm^−1^ corresponds to the –C = O stretching vibration of the ester group and the peaks at 1,454 and 1,360 cm^−1^ are assigned to the asymmetric and symmetric -CH_3_ deformation vibrations, respectively. The C = O bending appeared at 1,265 cm^−1^, the –C-O-C- stretching of the ester groups at 1,182 cm^−1^, the C-O stretching at 1,130 and 1,088 cm^−1^ and the -OH bending at 1,043 cm^−1^. It was also identified the peak at 872 cm^−1^, which corresponds to the –C-C- stretching of the amorphous phase (Muller et al., 2017).

No relevant variations in the chemical structure of the PLA were observed with the presence of the nanoparticles (LNP or CNC) or the AI in both monolayers (Figure 2B) and bilayers films (Figure 2C), even if few variations are found: in the case of monolayer films, peak at 872 cm^−1^ was not found in presence of nanofillers and AI, indicating a variation in the crystallization behavior of the films (Scaffaro and Lopresti, 2018), while the signal at 1,454 and 1,265 cm^−1^, respectively due to the CH_3_ bending and C-O stretching were less evident. A double peak was also observed at 1,044 cm^−1^, typically related to –OH bending (Scaffaro et al., 2016).

Only the characteristic peaks of the lignin nanoparticles were more visible in the PLA_3LNP/PLA_15UMB bilayer film, in details the peaks at 1,163, 1,055, and 1,025 cm^−1^, which correspond to the C-O-C symmetric and antisymmetric stretching, C-O stretching modes, C-H bond in guaiacyl ring have been detected (Wang et al., 2016; Hidayati et al., 2018).

### Morphological Characterization

**Figure 3:**
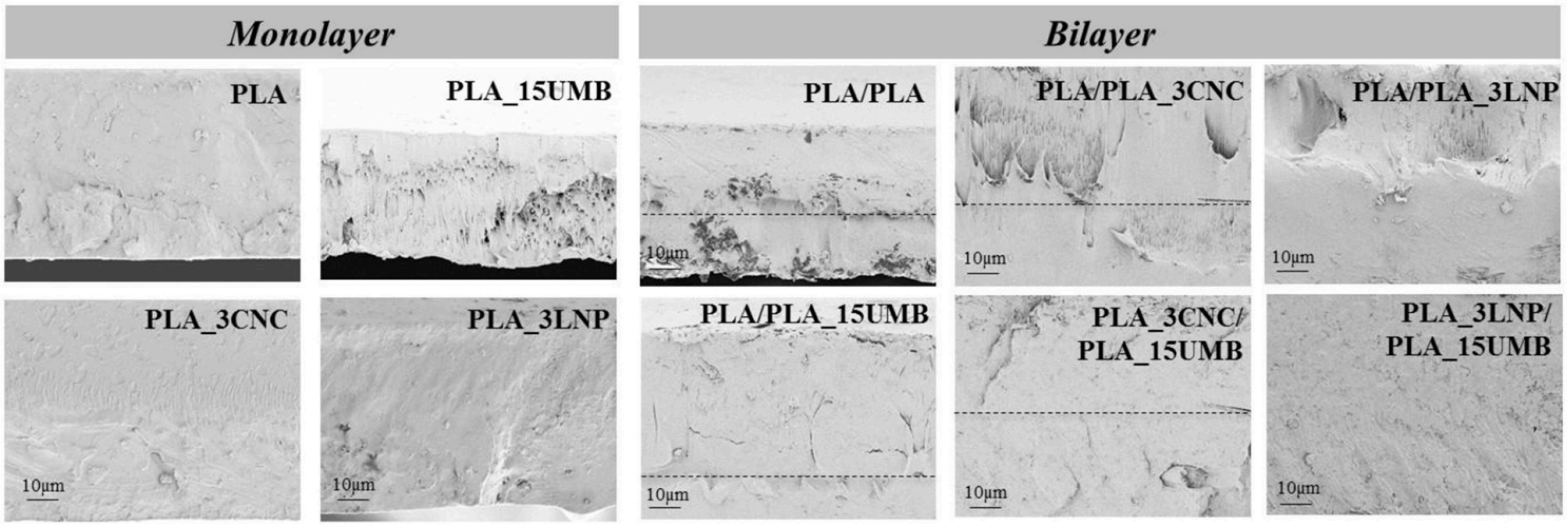
FESEM micrographs of cross-sections of PLA nanocomposites monolayer and bilayer films.

The fractured surfaces of the PLA based films studied by FESEM are shown in Figure 3. PLA film showed a smooth and homogeneous surface, which was in general preserved in the monolayer films containing the nanoparticles as well as the AI, highlighting a good interface interaction between LNP or CNC and PLA matrix. The surfaces appeared homogeneous, this phenomenon was due to a good dispersion of nanofillers induced by the adopted process. Furthermore, the monolayer film combined with CNC (PLA_3CNC) was characterized by a rougher fractured surface with respect of neat PLA and PLA_3LNP, underlining a more brittle tendency of this formulation (Yang et al., 2016b). The addition of UMB at 15% wt. affected the homogeneity and uniformity of PLA fractured surface, showing a ductile surface morphology for AI containing system. Nevertheless, better adhesion was observed in the bilayer films in presence of UMB: in PLA/PLA_15UMB, PLA_3CNC/PLA_15UMB, and PLA_3LNP/PLA_15UMB, thermo-compression leads to a good homogeneity of the films and complete adhesion of the two polymeric layers (Requena et al., 2016). In addition, the thickness of bilayer sheets was limited during thermo-compression to a different extent, depending on the film (Table 1). In fact, the overall measured thickness for bilayers was lower than the theoretical value (sum of each layer thickness). In case of film containing the AI, UMB incorporation promoted the flow of PLA layers, which suggests the UMB diffusion into the matrix, producing a plasticizing effect and greater thickness reduction (Muller et al., 2017).

**Table 1:** Thickness (mean value, μm) and color parameters for PLA (neat and nanocomposites) monolayer and bilayer films.

**Formulations**	**Thickness (μm)**	L^*^	a^*^	b^*^	ΔE^*^	**Gloss (°)**
White control		99.47 ± 0.00	−0.08 ± 0.01	−0.08 ± 0.01	–	121 ± 0
**MONOLAYER**						
PLA	53.9 ± 8.2	97.39 ± 0.04	−0.06 ± 0.00	0.20 ± 0.02	0.63 ± 0.04	254 ± 8
PLA_3CNC	118.8 ± 25.9	98.06 ± 0.10	−0.13 ± 0.02	0.70 ± 0.03	1.62 ± 0.07	129 ± 2
PLA_3LNP	140.0 ± 15.5	73.58 ± 2.920	5.41 ± 0.34	30.31 ± 1.77	40.32 ± 3.20	93 ± 3
PLA_15UMB	62.0 ± 8.4	85.34 ± 0.50	3.53 ± 0.15	18.79 ± 0.43	23.85 ± 0.66	37 ± 2
**BILAYER**						
PLA/PLA	81.5 ± 10.3	98.78 ± 0.05	−0.09 ± 0.00	0.26 ± 0.02	0.77 ± 0.05	163 ± 9
PLA/PLA_3CNC	127.1 ± 20.0	97.13 ± 0.40	−0.14 ± 0.03	1.88 ± 0.23	3.05 ± 0.44	135 ± 7
PLA/PLA_3LNP	119.3 ± 8.4	78.66 ± 1.39	4.09 ± 0.17	26.17 ± 1.25	33.76 ± 1.84	98 ± 4
PLA/PLA_15UMB	89.4 ± 11.5	85.46 ± 0.28	3.82 ± 0.11	18.59 ± 0.31	23.66 ± 0.42	45 ± 0
PLA_3CNC/PLA_15UMB	123.5 ± 17.3	88.86 ± 0.97	2.02 ± 0.19	13.56 ± 0.18	17.42 ± 0.74	71 ± 1
PLA_3LNP/PLA_15UMB	102.0 ± 18.7	52.95 ± 2.30	12.37 ± 0.50	32.29 ± 0.52	58.04 ± 1.84	40 ± 3

### Light Transmission and Color Properties

Optical properties are essential when a material is considered to be used for food packaging purposes and consumer acceptability is directly related to their color attributes. Color parameters of the different formulations are reported in Table 1. Pristine PLA showed a high L^*^ value representative of its brightness and transparency, while its colorless nature is consistent with a^*^ and b^*^ values close to zero. Similar results were obtained for PLA_3CNC film. However, luminosity (L^*^) value of the PLA_3LNP and PLA_15UMB was lower than that of the PLA film and the increment of the parameters a^*^ and b^*^ are indicative of a red-yellowish coloration, which is due to the typical color of LNP and AI. This tendency was also reflected in the significant value of the total color difference (ΔE) relative to the control. Bilayer films followed a similar tendency than the monolayer materials according the type of nanoparticle or AI added to the PLA. Then, PLA_3LNP/PLA_15UMB was the least transparent and the most yellowish film among the materials studied (Masek, 2015; Pagno et al., 2016). Mono and bilayer films showed also a lower gloss value than the pristine PLA film: the lowest value among the monolayer films was measured for the PLA_15UMB sample, due to the poorer dispersion of the AI in the PLA matrix. The results of the bilayer films are consistent with the corresponding monolayer ones and they are probably also influenced by the processing method used to mold the bilayers (Iiguez-Franco et al., 2012).

**Figure 4:**
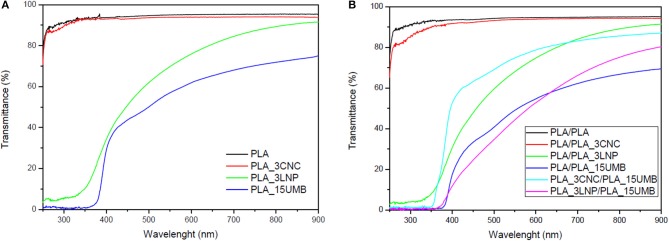
UV–vis spectra of PLA and PLA nanocomposites **(A)** monolayer films; **(B)** bilayer films.

UV-Vis characterization of the produced materials was also performed and related results for monolayer and bilayer films are reported in Figure 4. PLA film showed a high transparency, since it exhibited a high transmittance percentage in the entire measured wavelength region. The addition of LNP reduced the transmittance in the visible region, reaching rather null values at wavelength lower than 350 nm in the UV region, demonstrating the potential applicability of PLA_LNP as all-biomass packaging and coating systems characterized by exceptional transparency and UV-protection capability, mainly significant for light-sensitive products (Kim et al., 2017). A food packaging material is expected to be transparent to visible light and opaque in the UV region, in order to protect the food from the oxidative deterioration, discoloration and flavors losses causes by the UV radiation (Li et al., 2014).

The transparency of PLA_15UMB film is radically reduced above 350 nm, being this behavior induced by the presence of UMB that absorbs the light between 320 and 412 nm (Abu-Eittah and El-Tawil, 1985; Luzi et al., 2018b). Inversely, the addition of CNC did not modify the transparency of the PLA films. In a previous work it was found that PHB/CNC films are more transparent than films of pristine polymer (Seoane et al., 2017).

In bilayer films, PLA/PLA and PLA/PLA_3CNC showed a high light transmission as the respective monolayers did. On the other hand, the presence of an additional PLA layer in PLA/PLA_3LNP induced a further decrease of the signal in the visible region and UV protection substantially unchanged with respect of monolayer. The others bilayer formulations also behave rather transparent in the visible region and opaque in the UV section, which is an advantage for the possible application of the materials. The behavior was basically the same, with a general similar trend but a reduced intensity of the signal in of PLA/PLA_15UMB and PLA_3LNP/PLA_15_UMB, where LNP and AI acted in a synergic manner to modulate the transparency. Surprisingly, the combination of CNC and UMB in the bilayer system (PLA_3CNC/PLA_15UMB) induces significant improvements in terms of visible transparency, in accordance with results for color estimation (Table 1). Similar results were obtained by Aulin et al. (2013), where the polymer/NFC assembly with some minor nanofibrils aggregation resulting in surface light scattering and a slightly lower light transmittance and in a similar approach, by Halász et al. (2015), that proved retained transparency for PLA films coated with 4 layers of CNC in a layer-by-layer self-assembly methodology.

### Thermal Properties

**Figure 5:**
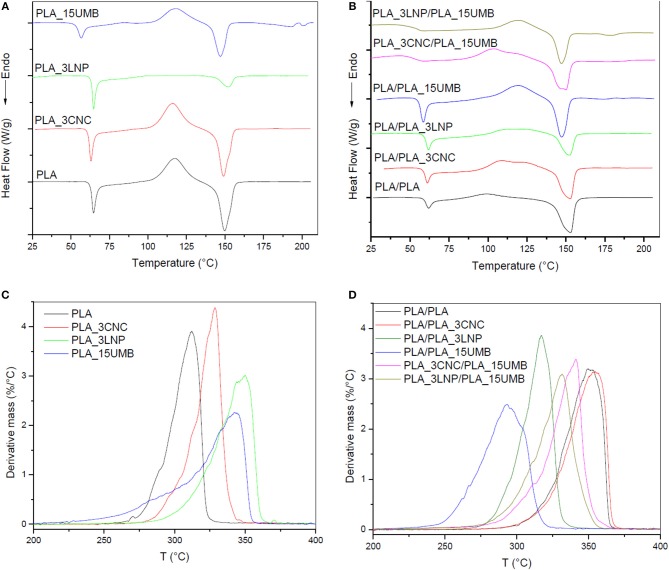
DSC (first heating scans) and derivative mass loss curves of PLA and PLA nanocomposites monolayer **(A–C)** and bilayer **(B–D)** films.

DSC tests of PLA-based mono and bilayer films were also carried out in order to investigate the role of AI and biobased nanofillers on polymer thermal characteristics. Figures 5A,B and Table 2 summarize the acquired data. No significant variations in T_g_ and T_m_ neither in the percentage of cristallinity were found with the addition of CNC or LNP to PLA matrix.

**Table 2:** Thermal properties from DSC first heating scans of PLA (neat and nanocomposites) monolayer and bilayer films.

**Formulation**	**Tg (°C)**	**Tcc (°C)**	**ΔHcc (J/g)**	**Tm (°C)**	**ΔHm (J/g)**	**Xc (%)**
**MONOLAYER**						
PLA	63	118	24	150	28	5
PLA_3CNC	62	116	28	149	30	2
PLA_3LNP	63	126	4	152	7	3
PLA_15UMB	55	84	1	147	26	12
**BILAYER**						
PLA/PLA	60	99	5	153	31	28
PLA/PLA_3CNC	60	108	17	158	28	12
PLA/PLA_3LNP	60	112	8	152	16	9
PLA/PLA_15UMB	57	119	20	147	24	6
PLA_3CNC/ PLA_15UMB	53	103	17	150	28	14
PLA_3LNP/ PLA_15UMB	55	120	13	147	17	7

Incorporation of amorphous lignin nanoparticles led to less crystalline regions established in the monolayer systems: in comparison with other biopolymer nanoparticles that they could facilitate the crystallization phenomenon through the nucleation phase, lignin nanoparticles are characterized by reduced contribution to the PLA crystal formation and growth, even though they were compatible with PLA (Kubo and Kadla, 2003). As evidenced by XRD analysis, the crystalline structure of the PLA_3LNP and PLA_3CNC system was nearly the same as that of neat PLA film.

However, a reduction in T_g_ and T_cc_, as well as an increment in the percentage of crystallinity, was observed in the PLA_15UMB film compared to neat PLA, probably due to a plasticizing effect of UMB on the PLA matrix (López-de-Dicastillo et al., 2012), in accordance to XRD and FTIR results.

In the case of bilayer films, a similar reduction in T_g_ was observed for the systems containing the AI.

Moreover, the thermogram of PLA/PLA_15UMB showed a delineated endothermic peak, respect to other systems, this phenomenon can be ascribed to the glass transition, which it is normally related to a stronger stress relaxation on heating, particularly in the presence of an ingredient with a plasticizing effect in the polymeric thin systems, in accordance to the results of Fortunati and co-authors in presence of limonene containing PLA films (Fortunati et al., 2014).

An evidence of the CNC pivot role in promoting the crystallization in multilayer films can be found in the reduced T_cc_ values, even in presence of a microscale AI (108 and 103°C, respectively for PLA/PLA_3CNC and PLA_3CNC/PLA_15UMB films): in addition, the combination of both UMB and CNC in the bilayer induced a further crystallization event (that can be identified in the shoulder peak at T = 120°C, coincident with the cold crystallization temperature of PLA/PLA_15UMB).

In order to investigate the presence of the additives and their behavior upon processing, thermal degradation analysis of the mono and bilayer films was performed. The derivative curves of residual mass vs. temperature are shown in Figures 5C,D for the mono and bilayer films, respectively.

PLA showed a single degradation process under nitrogen atmosphere, with a maximum degradation temperature (T_d_) at 305°C. It was observed that T_d_ values of the PLA-nanocomposites are higher than that of the PLA, due to the barrier effect of the nanoparticles. Lignin nanocomposites showed the highest increment of T_d_, which is consistent with its charring forming characteristics of polyphenolic LNP (Gupta et al., 2014), while CNC confirmed their ability to increase the thermal stability of PLA and slowing down the rate of thermal degradation (Fortunati et al., 2012; Khoo et al., 2016).

PLA_15UMB showed a reduced degradation rate, but an anticipated T_onset_, in comparison with PLA_3LNP and this behavior can be correlated to the thermo stability of the respective filler, as already evidenced by He et al. (2018) and Luzi et al. (2018b). PLA/PLA and PLA/PLA_3CNC bilayer films exhibited increased thermal stability compared to the corresponding monolayer films. This behavior could be related to the increment in the percentage of cristallinity of the samples, as it was observed by DSC (Table 2). While, the T_d_ of PLA_15UMB was higher than that of PLA/PLA_15UMB, probably due to a plasticizing effect of UMB and a diminution in the Xc in the bilayer system. The combined effect of CNC and UMB lead to a Td of 340°C which could be related to the UMB diffusion during thermo-compression, as explained previously by XRD, as well as to the increment of X_c_. Both mono and bilayers films completely degrade at the end of the test at 800°C.

### Mechanical Properties

**Figure 6:**
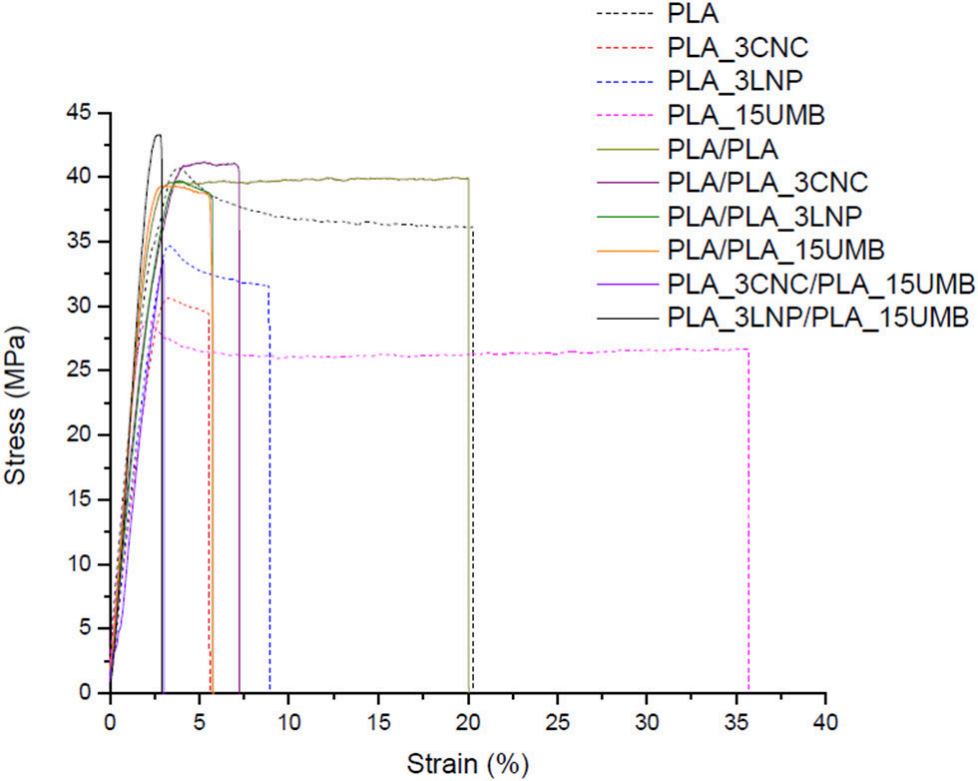
Stress-strain curves of PLA (neat and nanocomposites) monolayer and bilayer films.

Figure 6 shows the typical stress–strain curves of PLA based systems and the results of tensile test in terms of modulus, strength and elongation at break are listed in Table 3. Regarding tensile properties, the presence of 3% of nanoparticles provokes a decrease in the elongation at break of the PLA films respect to the neat polymer, which is a common trend observed for thermal processable nanocomposites (Yang et al., 2015a; Ferreira et al., 2018). However, no relevant increment in the modulus and tensile strength was observed with the addition of the nanoparticles. This behavior could be due to the poor dispersion of the nanoparticles reducing their interactions with the PLA matrix. A remarkable improvement in the elongation as break was achieved with the AI addition in the PLA film, which was not kept in the bilayer materials. The presence of both the nanoparticle and the AI in each film enhance the modulus and the tensile strength of the bilayer material, assuming a good interface adhesion controlling the bilayer resistance. If a probable use of these materials is as packaging, high elongation at break and enhanced mechanical resistance are necessary.

**Table 3:** Tensile properties [elastic modulus (E), tensile strength (TS) and elongation at break (ε)] of PLA (neat and nanocomposites) monolayer and bilayer films.

**Formulation**	**E (GPa)**	**TS (MPa)**	**ε (%)**
**MONOLAYER**			
PLA	1.4 ± 0.2	41.5 ± 3.9	16.2 ± 4.2
PLA_3CNC	1.2 ± 0.1	28.1 ± 4.0	4.9 ± 0.8
PLA_3LNP	1.3 ± 0.1	34.2 ± 1.5	8.4 ± 0.9
PLA_15UMB	1.2 ± 0.3	28.8 ± 9.3	37.0 ± 4.2
**BILAYER**			
PLA/PLA	1.6 ± 0.2	35.0 ± 6.2	16.4 ± 5.3
PLA/PLA_3CNC	1.4 ± 0.1	38.3 ± 2.9	5.6 ± 1.2
PLA/PLA_3LNP	1.5 ± 0.3	36.7 ± 4.1	6.3 ± 0.8
PLA/PLA_15UMB	1.6 ± 0.1	38.4 ± 2.2	4.5 ± 0.9
PLA_3CNC/ PLA_15UMB	2.1 ± 0.1	52.9 ± 2.2	2.9 ± 0.3
PLA_3LNP/ PLA_15UMB	2.0 ± 0.3	48.4 ± 4.4	3.0 ± 0.2

### Antioxidant Activity

**Figure 7:**
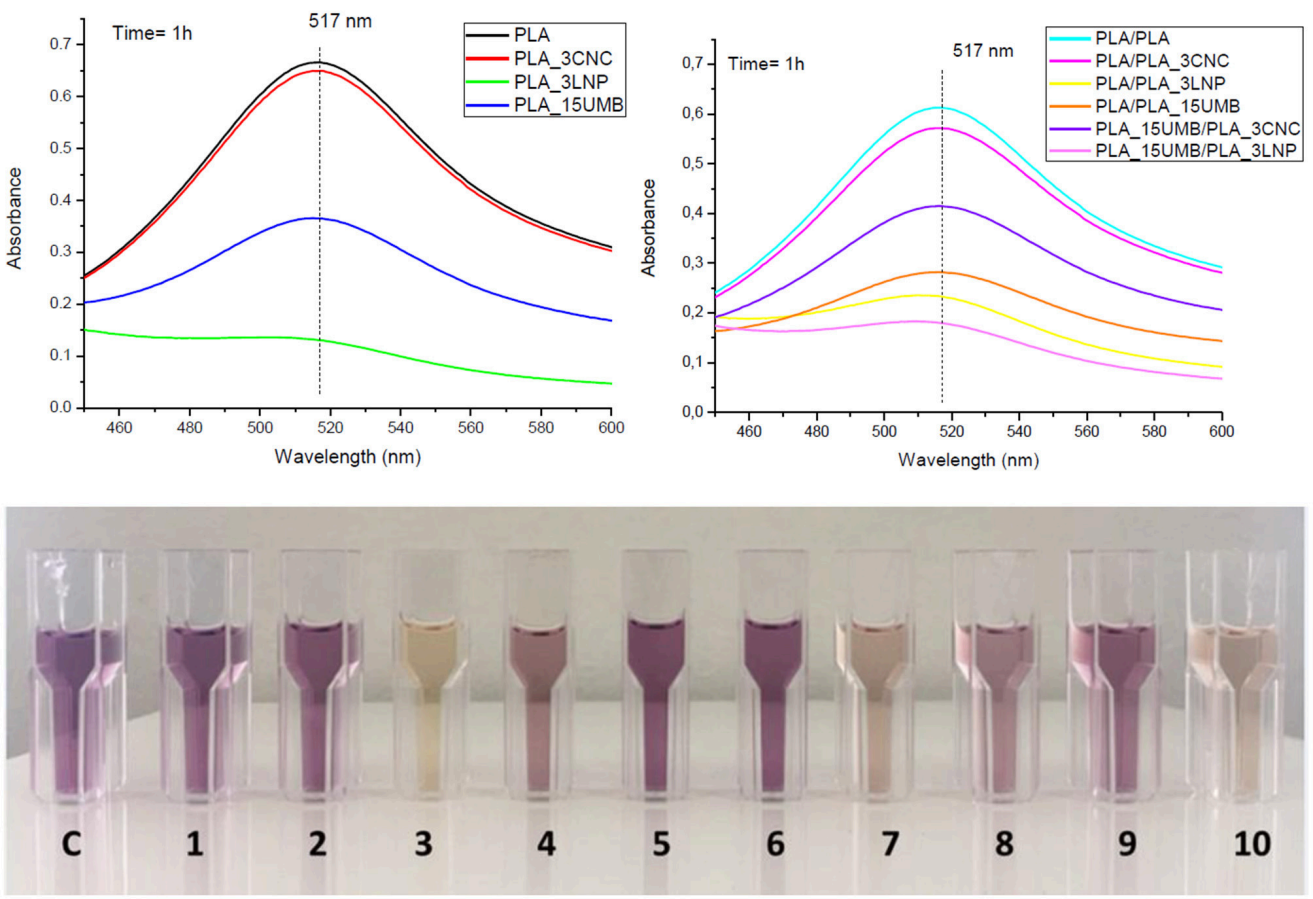
Antioxidant activities of PLA (neat and nanocomposites) monolayer and bilayer films evaluated by UV absorbance of DPPH at 517 nm monitored after 1 h and color variation of the DPPH methanol solution (C = control, 1 = PLA, 2 = PLA_3CNC, 3 = PLA_3LNP, 4 = PLA_15UMB, 5 = PLA/PLA, 6 = PLA/PLA_3CNC, 7 = PLA/PLA_3LNP, 8 = PLA/PLA_15UMB, 9 = PLA_15UMB/PLA_3CNC, 10 = PLA_15UMB/PLA_3LNP).

Antioxidant capacity of the mono and bilayer films was measured and expressed as radical scavenging activity (RSA). The values are listed in Table 4. Figure 7 shows the color variation of the DPPH methanol solution for PLA mono and bilayer neat and nanocomposites films.

**Table 4:** Radical scavenging activity (RSA) values for PLA (neat and nanocomposites) monolayer and bilayer films.

**Formulations**	**DPPH scavenging activity, RSA (%)**
**MONOLAYER**	
PLA	–
PLA_3CNC	2.5
PLA_3LNP	80.4
PLA_15UMB	45.2
**BILAYER**	
PLA/PLA	–
PLA/PLA_3CNC	6.8
PLA/PLA_3LNP	62.0
PLA/PLA_15UMB	54.0
PLA_3CNC/PLA_15UMB	32.3
PLA_3LNP/PLA_15UMB	70.7

*Time = 1 h*.

LNP produced the highest antioxidant activity in the monolayer formulations reaching a RSA value of 80%, as previously reported in Yang et al. (2018), while CNC did not show a significant radical scavenging activity in the PLA matrix. On the other hand, UMB was able to limit the oxidation by donating hydrogen from the phenolic hydroxyl groups, by formation of a stable end product, unable to initiate or propagate oxidation of the lipids (Murthy and Naidu, 2012). The same trend was maintained even in the case of bilayer films in presence of CNC, while LNP in the layered PLA showed reduced activity, due to a limited diffusion of these additives in thicker films. On the other hand, the incorporation of UMB in the bilayer PLA films induced the highest RSA among those materials, due to the synergistic effect of both additives. The presence of UMB causes a clear effect of RSA %: the highest effect is related by the incorporation of LNP respect to CNC (RSA (%): PLA_3CNC/PLA_15UMB = 32.3% and PLA_3LNP/PLA_15UMB = 70.7%), definitively higher than the corresponding PLA/PLA_3CNC = 6.8% and PLA/PLA_3LNP = 60.2% (López et al., 2007; Luzi et al., 2018b).

This phenomenon has been reported for polymeric films containing epicatechin, quercetin, catechin, caffeine, gallic acid, in which the antioxidant effect was proportional to the antioxidant content in the simulant. Similar behavior was observed by using extracts obtained from different varieties of grapes, the antioxidant activity increases with the concentration of the phenolic compounds (López-de-Dicastillo et al., 2010).

## Conclusions

Bilayer films based on PLA were obtained by extrusion followed by monolayer thermo-compression. CNC and LNP as well as an active ingredient (UMB) were added to the formulation in order to improve the PLA properties as food packaging material.

No significant variations in the chemical structure of PLA with the presence of the nanoparticles or UMB in both monolayers and bilayers films were observed by FTIR. Fractured surfaces of the PLA based films studied by FESEM revealed a homogeneous morphology with the nanoparticles addition while a less uniform surface was observed for UMB containing system. Additionally, a reduction in the bilayer thickness was observed compared to sum of each monolayer.

Brightness and transparency of PLA was not affected by the CNC addition, while LNP and UMB notably reduce the luminosity of both the mono and bilayer materials. However, the PLA_3CNC/PLA_15UMB bilayer system showed a notable transparency in the visible region. All the materials showed a good protection against UV light what is an advantage considering their use as food packaging.

A plasticizing effect of UMB on the PLA matrix was noticed in accordance with the T_g_ and T_cc_ reduction observed by DSC. This was in agreement to XRD and FTIR results. The percentage of PLA cristallinity was generally enhanced in the bilayer films respect to the monolayer ones which was also evidenced in the thermal resistance increment of those bilayer films. T_d_ of monolayer PLA-nanocomposites were higher than that of PLA due to the barrier effect of the nanoparticles, however UMB reduced the T_d_ due to its own lower thermo stability.

Regarding mechanical properties, monolayers showed an increment in the elongation at break compared to the bilayer systems, with a reduction in the modulus and tensile strength according to the relative cristallinity values of the samples. Moreover, a notable enhancement in the elongation at break was reached with the UMB addition to the PLA film due to the plasticizing effect of the AI.

LNP produced the highest antioxidant activity in the monolayer formulations while the combined incorporation of LNP and UMB in the bilayer PLA films induced the highest antioxidant activity due to the synergistic effect of both additives.

## Data Availability

All datasets generated for this study are included in the manuscript and/or the supplementary files.

## Author Contributions

MI, DP, FL, and FD contributed to conception and design of the study. MI, LM, and VC wrote the first draft of the manuscript. MI, DP, and FL wrote sections of the manuscript. All authors contributed to manuscript revision, read and approved the submitted version.

### Conflict of Interest Statement

The authors declare that the research was conducted in the absence of any commercial or financial relationships that could be construed as a potential conflict of interest.

## References

[B1] Abdelwahab M. A. Flynn A. Chiou B.-S. Imam S. Orts W. Chiellini E. (2012). Thermal, mechanical and morphological characterization of plasticized PLA–PHB blends. Polym. Degrad. Stab. 97, 1822–1828. 10.1016/j.polymdegradstab.2012.05.036

[B2] Abu-Eittah R. H. El-Tawil B. A. H. (1985). The electronic absorption spectra of some coumarins. A molecular orbital treatment. Can. J. Chem. 63, 1173–1179. 10.1139/v85-200

[B3] Arrieta M. P. López J. Ferrándiz S. Peltzer M. A. (2013). Characterization of PLA-limonene blends for food packaging applications. Polym. Test. 32, 760–768. 10.1016/j.polymertesting.2013.03.016

[B4] Arrieta M. P. López J. López D. Kenny J. M. Peponi L. (2016). Effect of chitosan and catechin addition on the structural, thermal, mechanical and disintegration properties of plasticized electrospun PLA-PHB biocomposites. Polym. Degr. Stabil. 132 (Suppl. C), 145–156. 10.1016/j.polymdegradstab.2016.02.027

[B5] ASTM D523 (2018). Standard Test Method for Specular Gloss.

[B6] Aulin C. Karabulut E. Tran A. Wgberg L. Lindstr T. (2013). Transparent nanocellulosic multilayer thin films on polylactic acid with tunable gas barrier properties. ACS Appl. Mater. Interfaces 5, 7352–7359. 10.1021/am401700n 23834391

[B7] Banerjee R. Verma A. K. Siddiqui M. W. (2017). Control of lipid oxidation in muscle food by active packaging technology, in Natural Antioxidants, eds Banerjee R. Verma A. K. Siddiqui M. W. (New York, NY: Apple Academic Press), 363–402.

[B8] Bassett K. H. Liang C. Y. Marchessault R. H. (1963). The infrared spectrum of crystalline polysaccharides. IX. The near infrared spectrum of cellulose. J. Polym. Sci. A General Papers 1, 1687–1692. 10.1002/pol.1963.100010520

[B9] Byun Y. Kim Y. T. Whiteside S. (2010). Characterization of an antioxidant polylactic acid (PLA) film prepared with a -tocopherol, BHT and polyethylene glycolusing film cast extruder. J. Food Eng. 100, 239–244.

[B10] Castro López M. M. López de Dicastillo C. López Vilariño J. M. González Rodríguez M. V. (2013). Improving the capacity of polypropylene to be used in antioxidant active films: incorporation of plasticizer and natural antioxidants. J. Agric. Food Chem. 61, 8462–8470. 10.1021/jf402670a 23941531

[B11] Cerruti P. Malinconico M. Rychly J. Matisova-Rychla L. Carfagna C. (2009). Effect of natural antioxidants on the stability of polypropylene films. Polym. Degrad. Stab. 94, 2095–2100. 10.1016/j.polymdegradstab.2009.07.023

[B12] Chaiwutthinan P. Pimpan V. Chuayjuljit S. Leejarkpai T. (2015). Biodegradable plastics prepared from poly (lactic acid), poly (butylene succinate) and microcrystalline cellulose extracted from waste-cotton fabric with a chain extender. J. Polym. Environ. 23, 114–125. 10.1007/s10924-014-0689-0

[B13] Chung Y.-L. Olsson J. V. Li R. J. Frank C. W. Waymouth R. M. Billington S. L. (2013). A renewable lignin–lactide copolymer and application in biobased composites. ACS Sustain. Chem. Eng. 1, 1231–1238. 10.1021/sc4000835

[B14] Domenek S. Louaifi A. Guinault A. Baumberger S. (2013). Potential of lignins as antioxidant additive in active biodegradable packaging materials. J. Polym. Environ. 21, 692–701. 10.1007/s10924-013-0570-6

[B15] Domínguez R. Barba F. J. Gómez B. Putnik P. Bursać Kovačevi,ć D. Pateiro M.. (2018). Active packaging films with natural antioxidants to be used in meat industry: a review. Food Res. Int. 113, 93–101. 10.1016/j.foodres.2018.06.073 30195551

[B16] El-Wakil N. A. Hassan E. A. Abou-Zeid R. E. Dufresne A. (2015). Development of wheat gluten/nanocellulose/titanium dioxide nanocomposites for active food packaging. Carbohydr. Polym. 124, 337–346. 10.1016/j.carbpol.2015.01.076 25839828

[B17] Fabra M. J. López-Rubio A. Lagaron J. M. (2016). Use of the electrohydrodynamic process to develop active/bioactive bilayer films for food packaging applications. Food Hydrocoll. 55, 11–18. 10.1016/j.foodhyd.2015.10.026

[B18] Fernandes E. M. Pires R. A. Mano J. F. Reis R. L. (2013). Bionanocomposites from lignocellulosic resources: properties, applications and future trends for their use in the biomedical field. Prog. Polym. Sci. 38, 1415–1441. 10.1016/j.progpolymsci.2013.05.013

[B19] Ferreira F. V. Dufresne A. Pinheiro I. F. Souza D. H. S. Gouveia R. F. Mei L. H. I. (2018). How do cellulose nanocrystals affect the overall properties of biodegradable polymer nanocomposites: a comprehensive review. Eur. Polym. J. 108, 274–285. 10.1016/j.eurpolymj.2018.08.045

[B20] Fortunati E. Armentano I. Zhou Q. Iannoni A. Saino E. Visai L. (2012). Multifunctional bionanocomposite films of poly(lactic acid), cellulose nanocrystals and silver nanoparticles. Carbohydr. Polym. 87, 1596–1605. 10.1016/j.carbpol.2011.09.066

[B21] Fortunati E. Luzi F. Fanali C. Dugo L. Belluomo M. G. Torre L. (2016a). Hydroxytyrosol as active ingredient in poly (vinyl alcohol) films for food packaging applications. J. Renew. Materials 5, 81–95. 10.7569/JRM.2016.634132

[B22] Fortunati E. Luzi F. Puglia D. Dominici F. Santulli C. Kenny J. M. (2014). Investigation of thermo-mechanical, chemical and degradative properties of PLA-limonene films reinforced with cellulose nanocrystals extracted from Phormium tenax leaves. Eur. Polym. J. 56, 77–91. 10.1016/j.eurpolymj.2014.03.030

[B23] Fortunati E. Puglia D. Luzi F. Santulli C. Kenny J. M. Torre L. (2013). Binary PVA bio-nanocomposites containing cellulose nanocrystals extracted from different natural sources: part I. Carbohydr. Polym. 97, 825–836. 10.1016/j.carbpol.2013.03.075 23911521

[B24] Fortunati E. Yang W. Luzi F. Kenny J. Torre L. Puglia D. (2016b). Lignocellulosic nanostructures as reinforcement in extruded and solvent casted polymeric nanocomposites: an overview. Eur. Polym. J. 80, 295–316. 10.1016/j.eurpolymj.2016.04.013

[B25] Ge Y. Wei Q. Li Z. (2014). Preparation and evaluation of the free radical scavenging activities of nanoscale lignin biomaterials. BioResources 9, 6699–6706. 10.15376/biores.9.4.6699-6706

[B26] Gorrasi G. Pantani R. (2013). Effect of PLA grades and morphologies on hydrolytic degradation at composting temperature: assessment of structural modification and kinetic parameters. Polym. Degrad. Stab. 98, 1006–1014. 10.1016/j.polymdegradstab.2013.02.005

[B27] Gupta A. K. Mohanty S. Nayak S. K. (2014). Synthesis, characterization and application of lignin nanoparticles (LNPs). Materials Focus 3, 444–454. 10.1166/mat.2014.1217

[B28] Halász K. Hosakun Y. Csóka L. (2015). Reducing water vapor permeability of poly (lactic acid) film and bottle through layer-by-layer deposition of green-processed cellulose nanocrystals and chitosan. Int. J. Polym. Sci. 2015:954290 10.1155/2015/954290

[B29] He X. Luzi F. Yang W. Xiao Z. Torre L. Xie Y. (2018). Citric acid as green modifier for tuned hydrophilicity of surface modified cellulose and lignin nanoparticles. ACS Sustain. Chem. Eng. 6, 9966–9978. 10.1021/acssuschemeng.8b01202

[B30] Hidayati S. Zuidar A. S. Satyajaya W. Retnowati D. (2018). Isolation and characterization of formacell Lignins from oil empty fruits bunches. IOP Conf. Ser. Mater. Sci. Eng. 344:012006 10.1088/1757-899X/344/1/012006

[B31] Iiguez-Franco F. Soto-Valdez H. Peralta E. Ayala-Zavala J. F. Auras R. Gámez-Meza N. (2012). Antioxidant activity and diffusion of catechin and epicatechin from antioxidant active films made of poly (L-lactic acid). J. Agric. Food Chem. 60, 6515–6523. 10.1021/jf300668u 22681400

[B32] Ittadwar P. A. Puranik P. K. (2017). Novel umbelliferone phytosomes: development and optimization using experimental design approach and evaluation of photo-protective and antioxidant activity. Int. J. Pharm. Pharm. Sci. 9, 218–228. 10.22159/ijpps.2017v9i1.14635

[B33] Jia S. Yu D. Zhu Y. Wang Z. Chen L. Fu L. (2017). Morphology, crystallization and thermal behaviors of PLA-based composites: wonderful effects of hybrid GO/PEG via dynamic impregnating. Polymers 9:10 10.3390/polym9100528 PMC6418665 30965832

[B34] Kalish J. P. Aou K. Yang X. Hsu S. L. (2011). Spectroscopic and thermal analyses of α′ and α crystalline forms of poly(l-lactic acid). Polymer 52, 814–821. 10.1016/j.polymer.2010.12.042

[B35] Kamal M. R. Khoshkava V. (2015). Effect of cellulose nanocrystals (CNC) on rheological and mechanical properties and crystallization behavior of PLA/CNC nanocomposites. Carbohydr. Polym. 123, 105–114. 10.1016/j.carbpol.2015.01.012 25843840

[B36] Khoo R. Z. Ismail H. Chow W. S. (2016). Thermal and morphological properties of poly (lactic acid)/nanocellulose nanocomposites. Procedia Chem. 19, 788–794. 10.1016/j.proche.2016.03.086

[B37] Kim Y. Suhr J. Seo H.-W. Sun H. Kim S. Park I.-K. (2017). All biomass and UV protective composite composed of compatibilized lignin and poly (Lactic-acid). Sci. Rep. 7:43596 10.1038/srep43596

[B38] Kubo S. Kadla J. F. (2003). The formation of strong intermolecular interactions in immiscible blends of poly (vinyl alcohol)(PVA) and lignin. Biomacromolecules 4, 561–567. 10.1021/bm025727p 12741770

[B39] Lee S. Y. Lee S. J. Choi D. S. Hur S. J. (2015). Current topics in active and intelligent food packaging for preservation of fresh foods. J. Sci. Food Agric. 95, 2799–2810. 10.1002/jsfa.7218 25892577

[B40] Li J.-H. Miao J. Wu J.-L. Chen S.-F. Zhang Q.-Q. (2014). Preparation and characterization of active gelatin-based films incorporated with natural antioxidants. Food Hydrocoll. 37, 166–173. 10.1016/j.foodhyd.2013.10.015

[B41] Liu Y. Gao W. Zhang C. Tang P. Zhao Y. Wu D. (2017). Sequential molecule-triggered-release system based on acetylated amylose helix aggregates. Chem. Commun. 53, 10680–10683. 10.1039/C7CC05783K 28905946

[B42] López P. Sánchez C. Batlle R. Nerín C. (2007). Development of flexible antimicrobial films using essential oils as active agents. J. Agric. Food Chem. 55, 8814–8824. 10.1021/jf071737b 17880148

[B43] López-de-Dicastillo C. Alonso J. M. Catalá R. Gavara R. Hernández-Muñoz P. (2010). Improving the Antioxidant protection of packaged food by incorporating natural flavonoids into ethylene–vinyl alcohol copolymer (EVOH) films. J. Agric. Food Chem. 58, 10958–10964. 10.1021/jf1022324 20879767

[B44] López-de-Dicastillo C. Gómez-Estaca J. Catal,á R. Gavara R. Hernández-Muñoz P. (2012). Active antioxidant packaging films: development and effect on lipid stability of brined sardines. Food Chem. 131, 1376–1384. 10.1016/j.foodchem.2011.10.002

[B45] Ludueña L. N. Fortunati E. Morán J. I. Alvarez V. A. Cyras V. P. Puglia D. (2015). Preparation and characterization of polybutylene-succinate/poly(ethylene-glycol)/cellulose nanocrystals ternary composites. J. Appl. Polym. Sci. 133:15 10.1002/app.43302

[B46] Luzi F. Fortunati E. Di Michele A. Pannucci E. Botticella E. Santi L.. (2018a). Nanostructured starch combined with hydroxytyrosol in poly(vinyl alcohol) based ternary films as active packaging system. Carbohydr. Polym. 193, 239–248. 10.1016/j.carbpol.2018.03.079 29773378

[B47] Luzi F. Fortunati E. Giovanale G. Mazzaglia A. Torre L. Balestra G. M. (2017). Cellulose nanocrystals from *Actinidia deliciosa* pruning residues combined with carvacrol in PVA_CH films with antioxidant/antimicrobial properties for packaging applications. Int. J. Biol. Macromol. 104, 43–55. 10.1016/j.ijbiomac.2017.05.176 28587959

[B48] Luzi F. Puglia D. Dominici F. Fortunati E. Giovanale G. Balestra G. M. (2018b). Effect of gallic acid and umbelliferone on thermal, mechanical, antioxidant and antimicrobial properties of poly (vinyl alcohol-co-ethylene) films. Polym. Degrad. Stab. 152, 162–176. 10.1016/j.polymdegradstab.2018.04.015

[B49] Martínez-Abad A. Ocio M. J. Lagaron J. M. (2014). Morphology, physical properties, silver release, and antimicrobial capacity of ionic silver-loaded poly(l-lactide) films of interest in food-coating applications. J. Appl. Polym. Sci. 131:21.

[B50] Masek A. (2015). Flavonoids as natural stabilizers and color indicators of ageing for polymeric materials. Polymers 7, 1125–1144. 10.3390/polym7061125

[B51] Mazimba O. (2017). Umbelliferone: sources, chemistry and bioactivities review. Bull. Facul. Pharm. Cairo Univ. 55, 223–232. 10.1016/j.bfopcu.2017.05.001

[B52] Muller J. González-Martínez C. Chiralt A. (2017). Poly(lactic) acid (PLA) and starch bilayer films, containing cinnamaldehyde, obtained by compression moulding. Eur. Polym. J. 95, 56–70. 10.1016/j.eurpolymj.2017.07.019

[B53] Murthy P. S. Naidu M. M. (2012). Recovery of phenolic antioxidants and functional compounds from coffee industry by-products. Food Bioprocess Technol. 5, 897–903. 10.1007/s11947-010-0363-z

[B54] Nair S. S. Sharma S. Pu Y. Sun Q. Pan S. Zhu J. Y.. (2014). High shear homogenization of lignin to nanolignin and thermal stability of nanolignin-polyvinyl alcohol blends. ChemSusChem 7, 3513–3520. 10.1002/cssc.201402314 25319811

[B55] Pagno C. H. de Farias Y. B. Costa T. M. H. de Oliveira Rios A. Flôres S. H. (2016). Synthesis of biodegradable films with antioxidant properties based on cassava starch containing bixin nanocapsules. J. Food Sci. Technol. 53, 3197–3205. 10.1007/s13197-016-2294-9 27784914 PMC5055884

[B56] Peelman N. Ragaert P. De Meulenaer B. Adons D. Peeters R. Cardon L. (2013). Application of bioplastics for food packaging. Trends Food Sci. Technol. 32, 128–141. 10.1016/j.tifs.2013.06.003

[B57] Piñeros-Hernandez D. Medina-Jaramillo C. López-Córdoba A. Goyanes S. (2017). Edible cassava starch films carrying rosemary antioxidant extracts for potential use as active food packaging. Food Hydrocoll. 63, 488–495. 10.1016/j.foodhyd.2016.09.034

[B58] Pracella M. Haque M. M.-U. Puglia D. (2014). Morphology and properties tuning of PLA/cellulose nanocrystals bio-nanocomposites by means of reactive functionalization and blending with PVAc. Polymer 55, 3720–3728. 10.1016/j.polymer.2014.06.071

[B59] Requena R. Jiménez A. Vargas M. Chiralt A. (2016). Poly [(3-hydroxybutyrate)-co-(3-hydroxyvalerate)] active bilayer films obtained by compression moulding and applying essential oils at the interface. Polym. Int. 65, 883–891. 10.1002/pi.5091

[B60] Rexali A. (2007). FTIR and FT-raman spectral investigation of 7-hydroxycoumarin. Cauvery Res. J. 1, 75–78.

[B61] Richter A. P. Brown J. S. Bharti B. Wang A. Gangwal S. Houck K.. (2015). An environmentally benign antimicrobial nanoparticle based on a silver-infused lignin core. Nat. Nanotechnol. 10:817. 10.1038/nnano.2015.141 26167765

[B62] Scaffaro R. Lopresti F. (2018). Processing, structure, property relationships and release kinetics of electrospun PLA/Carvacrol membranes. Eur. Polym. J. 100, 165–171. 10.1016/j.eurpolymj.2018.01.035

[B63] Scaffaro R. Lopresti F. Botta L. Maio A. (2016). Mechanical behavior of polylactic acid/polycaprolactone porous layered functional composites. Composit. B Eng. 98, 70–77. 10.1016/j.compositesb.2016.05.023

[B64] Seoane I. T. Cerrutti P. Vázquez A. Manfredi L. B. Cyras V. P. (2017). Polyhydroxybutyrate-based nanocomposites with cellulose nanocrystals and bacterial cellulose. J. Polym. Environ. 25, 586–598. 10.1007/s10924-016-0838-8

[B65] Wang C. Xiong Y. Fan B. Yao Q. Wang H. Jin C.. (2016). Cellulose as an adhesion agent for the synthesis of lignin aerogel with strong mechanical performance, Sound-absorption and thermal Insulation. Sci. Rep. 6:32383. 10.1038/srep32383 27562532 PMC5387396

[B66] Yang H. Wang J. Yang F. Chen M. Zhou D. Li L. (2016a). Active packaging films from ethylene vinyl alcohol copolymer and clove essential oil as shelf life extenders for grass carp slice. Pack. Technol. Sci. 29, 383–396. 10.1002/pts.2215

[B67] Yang W. Fortunati E. Bertoglio F. Owczarek J. S. Bruni G. Kozanecki M.. (2018). Polyvinyl alcohol/chitosan hydrogels with enhanced antioxidant and antibacterial properties induced by lignin nanoparticles. Carbohydr. Polym. 181, 275–284. 10.1016/j.carbpol.2017.10.084 29253973

[B68] Yang W. Fortunati E. Dominici F. Giovanale G. Mazzaglia A. Balestra G. M. (2016b). Synergic effect of cellulose and lignin nanostructures in PLA based systems for food antibacterial packaging. Eur. Polym. J. 79, 1–12. 10.1016/j.eurpolymj.2016.04.003

[B69] Yang W. Fortunati E. Dominici F. Kenny J. M. Puglia D. (2015a). Effect of processing conditions and lignin content on thermal, mechanical and degradative behavior of lignin nanoparticles/polylactic (acid) bionanocomposites prepared by melt extrusion and solvent casting. Eur. Polym. J. 71, 126–139. 10.1016/j.eurpolymj.2015.07.051

[B70] Yang W. Kenny J. M. Puglia D. (2015b). Structure and properties of biodegradable wheat gluten bionanocomposites containing lignin nanoparticles. Ind. Crops Prod. 74, 348–356. 10.1016/j.indcrop.2015.05.032

